# Diffusion-Weighted MRI for the Assessment of Liver Fibrosis: Principles and Applications

**DOI:** 10.1155/2015/874201

**Published:** 2015-03-19

**Authors:** Stefano Palmucci, Giuseppina Cappello, Giancarlo Attinà, Giovanni Fuccio Sanzà, Pietro Valerio Foti, Giovanni Carlo Ettorre, Pietro Milone

**Affiliations:** Radiodiagnostic and Radiotherapy Unit, University Hospital “Policlinico-Vittorio Emanuele”, 95123 Catania, Italy

## Abstract

The importance of an early identification of hepatic fibrosis has been emphasized, in order to start therapy and obtain fibrosis regression. Biopsy is the gold-standard method for the assessment of liver fibrosis in chronic liver diseases, but it is limited by complications, interobserver variability, and sampling errors. Several noninvasive methods have been recently introduced into clinical routine, in order to detect liver fibrosis early. One of the most diffuse approaches is represented by diffusion-weighted liver MRI. In this review, the main technical principles are briefly reported in order to explain the rationale for clinical applications. In addition, roles of apparent diffusion coefficient, intravoxel incoherent motion, and relative apparent diffusion coefficient are also reported, showing their advantages and limits.

## 1. Introduction

Several chronic hepatic diseases may develop cirrhosis in the liver parenchyma. Hepatic steatosis, iron overload, autoimmune hepatitis, chronic viral hepatitis, sclerosing biliary cholangitis, alcohol, and drugs represent the most frequent causes of liver cirrhosis. All these chronic diseases, after an early phase of inflammation, lead to parenchymal fibrosis, which plays an important role in the development of cirrhosis [1].

Fibrogenesis has been defined as a “wound-healing response that engages a range of cell types and mediators to encapsulate injury” [2]. It consists of a progressive deposition of extracellular matrix proteins, which reduces widening of interstitial spaces and creates distortion of normal hepatic architecture [3]. It has been widely accepted that early recognition of fibrosis is crucial for preventing development of chronic parenchymal disease. New experimental treatments have emphasized the importance of an early identification of fibrogenesis, in order to start therapy and obtain fibrosis regression [4–6].

Biopsy is the gold-standard modality for assessing the degree of fibrosis and for evaluating necrosis or inflammation. However, it is affected by many complications, including bleeding, pneumothorax, and procedure-related death, and could be limited by interobserver variability and sampling errors [3, 7–10]. In addition, liver biopsy is not used in the management of disease, especially when we have to repeat the examination after a short interval of time, as reported by Kim et al. [3].

For this reason, in the past years many noninvasive tests and diagnostic examinations have been introduced into clinical routine in order to detect liver fibrosis early.

The collection of serum markers of fibrosis, namely, fibrotest/fibrosure, has been widely used in the assessment of hepatic fibrosis [3, 11–14]. Serum levels of aptoglobulin, *α*2-macroglobulin, *γ*-globulin, *γ*-glutamyl transferase, apolipoprotein, and total bilirubin indicate a score of hepatic fibrosis with relatively good accuracy [12–14].

Other noninvasive modalities include Transient Elastography (TE), Acoustic Radiation Force impulse Imaging (ARFI), Real-Time Elastography (RTE), and Magnetic Resonance Elastography (MRE). Particularly, measurement using TE has been routinely introduced into the assessment of liver fibrosis [15, 16]. This technique is based on the measurement of liver hardness and stiffness. More specifically, a FibroScan test using TE measures the velocity of a vibration wave produced by an ultrasonography-like probe [17]. The time required to reach a certain depth of parenchyma from skin is conditioned by liver stiffness and hardness, which are related to the degree of fibrosis developed. The fibrotic burden is large if the waves rapidly propagate [17–19].

Also, MR with diffusion-weighted sequences has been used for detection and quantification of hepatic fibrosis. Its introduction into liver MRI has fuelled high expectations with several encouraging studies [7]. In the assessment of diffuse but also of focal parenchymal disease, diffusion-weighted imaging (DWI) is currently one of the most important added values of liver MRI. In particular this review focuses on the main technical principles and clinical applications of diffusion-weighted MRI in the assessment of liver fibrosis.

## 2. Assessment of Hepatic Fibrosis Using Diffusion-Weighted MRI

### 2.1. DWI: Technical Principles

Diffusion-weighted imaging has been routinely introduced into liver MRI protocol [20]. Several studies, in the past fifteen years, have investigated its contribution in the assessment of diffuse and focal hepatic disease [7, 20, 21]. Important researches have evaluated DWI capability in the quantification of hepatic fibrosis in chronic liver disease [7, 22, 23], in characterization and detection of focal liver lesions [24–26], and in monitoring response to treatment in oncological patients [27, 28].

Diffusion imaging is based on the sensitivity of MRI to motion [29]. It consists of spin-echo sequence where the main 180° focalization pulse is preceded and followed by two additional gradient pulses [30–33]. Proton response to these gradient pulses is strongly related to their movements, which generally follow a Brownian motion. Applying the first gradient field before 180° refocusing RF pulse, protons develop a phase shift. For static molecules, the second gradient pulse will be able to compensate the phase shift produced by the first one; no additional shift is generated from movement. The refocusing introduced by the second gradient will be visible on MR diffusion images as high signal.

In case of molecules that move in the direction of the gradients, the phase shift created by the first gradient will not be rephrased by the second gradient. In addition, if a net phase shift is observable for molecules with a certain degree of motion, the different phase shifts in case of Brownian motions—as reported in literature—“interfere with each other, resulting in imperfect refocusing of echo” [31]. Thus, the second gradient field, applied after 180° focalization pulse, is unable to obtain a full compensation of phase acquired after first gradient, for the fact that molecules proceed with different directions in a Brownian modality.

### 2.2. Apparent Diffusion Coefficient

Diffusion-weighted pulse sequences need relatively long echo times (between 60 and 120 ms) for the application of the two diffusion gradients [34]. Thus, DW images are also intrinsically T2-weighted. Consequently, areas of high intensity on T2-weighted images may lead to high signal on DW images, even if the diffusion of water molecules in tissue may not be reduced. This imaging feature is called “T2 shine-through effect” [35]. To remove the T2-effect, it is recommended to acquire images with at least two *b*-values; the first one with a *b*-value of 0 and the second one with a higher *b*-value [36]. The greater the strength and timing of the gradients (collectively expressed by the *b*-value) are, the greater the sensitivity of sequence to microscopic diffusion is [36].

Apparent diffusion coefficient (ADC) is the main quantitative parameter used for quantifying proton diffusion motions in tissues and it is estimated using images acquired with two different *b*-values.

ADC is calculated with the following formula:

(1) $$\begin{array}{c} \text{ADC}=\frac{\ln\left(S0/S1\right)}{b}, \end{array}$$

where *S*0 is the signal intensity with *b* = 0, *S*1 is the signal intensity after the application of a given *b* gradient, and *b* is the strength of the applied gradient [21].

It is important to say that the smaller the maximum *b*-value used, the greater the ADC values: that is caused by the contribution of intravoxel incoherent motion (IVIM) effects (e.g., capillary perfusion and flow phenomena), which are more than diffusion. For higher *b*-values (≥300 s/mm^2^), “perfusion effects are cancelled out,” whereas ADCs obtained using very low *b*-values “could be increased by capillary perfusion” [24]. Therefore, maximum *b*-values, also greater than 800 sec/mm^2^ in Yamada's opinion, are necessary in order to reduce intravoxel incoherent motion effects [37].

In any case, it is important to remember that at higher *b*-values, images become noisier, so a compromise is often required; higher *b*-values give ADC information; lower *b*-values improve lesions detection. For these reasons, multiple *b*-values are usually performed [38].

An ADC map, which is a sort of subtraction image between sequences acquired with given *b*-value and sequences acquired with *b*-value of 0, can be used to exclude “T2 shine through effect.” Lesions showing hyperintense signal on ADC maps generally have increased diffusivity and high ADC values, whereas lesions with low signal on ADC maps have restricted diffusivity and low ADC values. The ADC can also be calculated using a multi-*b* analysis by fitting an exponential function to the measured signal intensities on multi-*b* acquired images [34].

### 2.3. DWI: Rationale for Use

In diffusion images, tissues containing molecules with high degree of movement and diffusion will be represented as dark areas of low signal, whereas tissues in which protons are unable to move around freely will have high signals [29].

Molecular diffusivity is conditioned by widening of interstitial spaces. Some clinical conditions could modify interstitial spaces, and water molecules could become unable to process randomly. Main diseases characterized by interstitial spaces narrower than normal are ischemic injury, tumour, abscess, haemorrhage/hematoma, and parenchymal fibrosis [39].

More specifically, ischemia reduces activity of cellular transporters along the cellular wall, causing increase in size of cells and contraction of interstitial spaces. Among neurological applications, DWI was the first to be introduced in the early assessment of cerebral stroke [40].

Also, a tumour could decrease Brownian motions, because of anarchic cell proliferation and hypercellularity. Anarchic proliferation reduces interstitial space and leads progressively to compression of small vessels, resulting in ischemic changes in the tissue. In view of these considerations, on diffusion-weighted images metastatic lesions reproduce different signal intensity on the basis of their cellularity [41]. Well-differentiated adenocarcinomas appear as hypointense lesions, whereas small and large cells neuroendocrine tumours show hyperintense signal [41].

Finally, several authors have postulated that fibrosis could also decrease the width of interstitial spaces. Fibrotic tissues generally develop as a consequence of chronic inflammation, with narrowing of interstitial spaces, and consequently proton motion reduction [42–45].

### 2.4. Diffusion-Weighted Sequences

DW imaging of the liver is influenced by motion, caused by breathing and cardiac pulsations, and lower T2 signal (liver is a tissue with intrinsically lower T2 signal, such as muscle) [46]. Thus, a different technique has been proposed for improving diffusion image quality and precision of ADC measurement.

Liver MRI protocol for assessing hepatic fibrosis is usually based on Single-Shot Echo Planar Imaging (SSEPI), which may be acquired in breath-hold modality, in respiratory-triggered or echo navigator modality, or also in free-breathing technique. Several studies have compared image quality and ADC reproducibility and repeatability among the different acquisition techniques mentioned.

Breath-hold SSEPI sequences are faster than triggered or free-breathing sequences and generally permit evaluation of the whole liver in one or two 20–30-second acquisitions. The short acquisition time reduces artifacts due to macroscopic physiological motions (respiratory, peristalsis). On the other hand, the higher acquisition speed causes low spatial resolution and signal-to-noise ratio (SNR), especially when images are acquired using higher *b*-values. They could also exhibit higher sensitivity to magnetic susceptibility artifacts caused by tissue/air interface [20, 47].

Respiratory-triggered DWI acquisitions require respiratory synchronization with patients' breath, generally obtained by placing a “respiratory” belt around their abdomen, and imaging data are usually acquired during end expiration phase. Respiratory-triggered sequences allow for high-quality images in patients with low-compliance for the exam, or in patients unable to maintain a breath-hold during the sequence acquisition [48, 49], at the expense of increased acquisition time [47]. For respiratory-triggered imaging, prospective acquisition correction technique (PACE) with a navigator sequence has been introduced, in order to better synchronize acquisition with patients' breath. This technique interleaves the imaging sequence with a navigator sequence [50]; the navigator is placed across the diaphragm [51]. It removes patients motion using a “real-time navigator pulse to trigger acquisition” at a specific point of respiratory cycle [50].

Free-breathing echo planar sequences could require a variable time for their acquisition; generally, many articles report about 3–6 minutes for whole liver evaluation [47]. These sequences may be performed when patients are not able to maintain breath-hold during the examination, due to the coexistence of respiratory or cardiac problems. Some patients with nonalcoholic steatohepatitis (NASH) could also be affected by obesity, with reduced pulmonary capacity. Long-time acquisition of free-breathing sequences is generally needed to improve the signal-to-noise ratio. Free-breathing diffusion images are slightly affected by cyclical respiration, because as reported by Kwee et al. “it is considered a coherent motion, where the acquired phase shift due to respiratory motion is equal in each phase-encoding step and then it does not result in additional signal attenuation” [52]. However, image blurring is not associated with breath-hold or respiratory triggered sequences. In view of these considerations, high signal averages or numbers of excitations are generally used for free-breathing diffusion sequences to increase the signal-to-noise ratio and reduce artifacts on images [52].

The efficacy of triggered sequences has been well documented in many articles published in literature. Naganawa et al. compared ADC values obtained with respiratory triggering and free-breathing [53]. They found higher ADC values in free-breathing sequences in the right lobe, suggesting that ADC values are influenced by respiratory motions. They also found that, in left lobe, ADC values are about the same in both types of sequence, supporting the fact that motion artifacts in the left lobe are mostly provoked by cardiac pulse [53]. Also Bruegel et al. suggest a pulse triggering in order to reduce cardiac motion artifacts [24].

Mürtz et al. also analyzed the influence of pulsatile motions on diffusion sequences and suggested acquiring data triggered by the cardiac cycle, in order to avoid data acquisition in different phases of the cardiac cycle [46]. The authors found that ADC values without triggering were artificially higher than those obtained with cardiac triggering [46]. However, simultaneous use of both respiratory and cardiac triggers make the sequence too long; thus Nasu et al. suggest use of an increased number of excitations, in order to increase the possibility of data acquisition during diastole, using only respiratory trigger [54].

Sandberg et al. compared respiratory triggered and breath-hold sequences and found better image quality for respiratory triggered DW-SSEPI [55].

Nasu et al. compared DW images acquired in free-breathing modality and with respiratory trigger; higher ADC measurement accuracy was reported using respiratory trigger [56].

Also, Bruegel et al. suggested use of DW-SSEPI using navigator-controlled respiratory triggering and parallel acquisition techniques in order to acquire high-quality diffusion-weighted images within a relatively short acquisition time (4–6 minutes) [24].

Taouli et al. evaluated the usefulness of navigator echo technique for triggered diffusion sequences; they found improvement in image quality and ADC measurement compared with standard breath-hold sequences. Of course, better image quality “is offset by longer acquisition time” [51].

In opposition to Taouli, Kwee et al. found higher ADC values with triggered acquisition [57], but they did not use a navigator echo.

In addition, respiratory triggered sequences could have artifacts, in particular a “pseudo-anisotropy artifact,” which induces errors in ADC measurement [54]: it takes its origin “in localized hepatic movements, such as extension, contraction and rotation.” This thesis is supported by the fact that pseudo-anisotropy artifact is more frequent in noncirrhotic livers. However, in their series this artifact did not interfere with diagnostic image interpretation [54].

Lastly, a degree of ADC variability is also present across different MR platforms, depending on the coil systems, imagers, vendors, and field strengths used for MR imaging, with an intervendor ADC measurement variability of 7% [58].

### 2.5. Quantification of Fibrosis: ADC

According to results published by one of the most important studies in this field [7], DWI was considered a “valid non-invasive method to predict the presence of moderate or advanced fibrosis.” Analyzing the study by Taouli et al., a total of 23 patients with chronic hepatitis and 7 healthy volunteers were examined using a 1.5-Tesla MR scanner [7]. Fibrosis was scored on a five-point scale: stage 0 = no fibrosis; stage 1 = portal fibrosis; stage 2 = periportal fibrosis; stage 3 = septal fibrosis; stage 4 = cirrhosis. ADC measurements were obtained by sampling different regions in the liver parenchyma and a Mann-Whitney *U* test was performed to predict the stage of fibrosis on the basis of ADC measurement. The authors found “lower hepatic ADCs in stage 2 or greater versus stage 1 or less fibrosis, and stage 3 or greater versus stage 2 or less fibrosis” [7]. ADC was a significant predictor of stage 2 or greater and stage 3 or greater fibrosis, with areas under the curve of 0.896 and 0.896. Moreover, a significant trend between degree of fibrosis and ADC values was reported; lower ADC values were associated to advanced fibrosis, whereas higher values were related to a mild degree of parenchymal fibrosis [7]. In a work by Bonekamp et al., significant differences were also reported comparing METAVIR stages F0 versus F1–4, F0-1 versus F > 1, F0–2 versus F3-4, and F0–3 versus F4 [Bonekamp]; a “substantial overlap” is also reported among ADC values [59].

However, a recent paper by Sandrasegaran et al. has investigated the usefulness of ADC in distinguishing different stages of fibrosis according to the METAVIR classification [60]. Mean ADCs were in fact calculated among different classes of fibrosis—including F0, F1, F2, F3, and F4 METAVIR stages. Contrarily to the previous study, ADC means was statistically different only between F0 and F4 classes, whereas no statistical difference was observed between patients with F2 stage or higher and patients with lower stages of fibrosis. The results obtained by Sandrasegaran et al. seem to challenge again the role of DWI in the evaluation of fibrosis [60].

In addition, a noninvasive method for evaluation of hepatic fibrosis should be reliable and able to completely substitute liver biopsy. The crucial point is that clinicians have to identify F2 stage of fibrosis early, because antifibrotic treatment is generally required for patients with moderate to advanced stage of fibrosis (F2 to F4). On the basis of their results, Sandrasegaran et al. conclude that DWI with current scanners is not reliable enough to replace liver biopsy [60].

### 2.6. Quantification of Fibrosis: Intravoxel Incoherent Motion (IVIM)

IVIM analysis means the “microscopic translational motions that occur in each image voxel” [61]. However, DWI imaging based on a monoexponential analysis could be limited, because it estimates molecular diffusion and the contribution of the perfusion effect. Perfusion is due to the random configuration of capillary network in each voxel. To separate diffusion and perfusion a biexponential model is required, with a multi-*b* fitting analysis.

Fifty-five patients with chronic liver disease were studied by diffusion-MRI using a 3-Tesla scanner [59]. IVIM-DWI was performed to evaluate the presence of hepatic fibrosis; true diffusion coefficient (*D*_*t*_), pseudo-diffusion coefficient (*D*_*p*_), perfusion fraction (*f*), and ADC total were calculated.

As reported by Yoon et al., all parameters “showed a significant correlation with the hepatic fibrosis stages,” and “were significantly higher in F0 to F1 than F4 (*P* < 0.05)” [62]. On the basis of their results, pseudo-diffusion coefficient was the best diagnostic tool in differentiating between *a* ≥ F2 and a F0 to F1 degree of parenchymal fibrosis [62], showing greater diagnostic accuracy than ADC. Thus, an IVIM-DWI analysis was suggested for the assessment of hepatic fibrosis.

In a study by Pasquinelli et al., healthy volunteers and patients with chronic hepatic disease were classified into four groups on the basis of their METAVIR score and their Child-Pugh score [23]: group 1 included all healthy volunteers, group 2 F0–F2 patients, group 3 F3-F4 Child AB patients, and finally group 4 patients having F4 Child CD score.

ADC, diffusion (*D*) values, and perfusion parameters were calculated. ADC mean values progressively decreased from first to fourth group, with no statistical difference observed among groups. A wide overlap was reported for ADC values, and according to results obtained in the study, “stratification of patients with chronic liver disease for clinical purposes cannot be done with DWI” [23].

### 2.7. Quantification of Fibrosis: Variability of Measurement

Considering results of the mentioned studies, the diagnostic capability of DWI in the evaluation of hepatic fibrosis seems to be controversial, with some papers not considering DWI a valid diagnostic tool able to replace liver biopsy. In fact, many limitations have been described in liver MRI protocol. First of all, ADC repeatability and reproducibility in liver parenchyma have been evaluated in many studies, with some controversial results [63]. In a study performed by Colagrande et al., four different ROIs (Regions Of Interest) were adopted to calculate ADC of the liver parenchyma: ROIs of 70% and 30% of the liver volume, 4%-one-ROI-per-segment, and 4%-one-ROI-per-slice in the right-lobe. Authors reported that repeatability was “acceptable” for all methods, but reproducibility was low, with ICC ≤ 0.45; for a large number of patients enrolled in their study (about 50%) it was impossible to obtain measurements in the left hepatic lobe [63].

Sampling parenchyma of left hepatic lobe could be very difficult due to the presence of magnetic susceptibility or motion artifacts in these locations, and even if the introduction of triggered-acquisition increases image quality, the evaluation of these areas could be limited [20].

Another important limitation for quantitative assessment of liver parenchyma is the possibility of magnetic susceptibility artifacts caused by colonic loop, often located very close to the caudal portion of the right liver. They reduce image quality on diffusion images, with low signal intensity of parenchyma and loss of liver profile [20].

### 2.8. Quantification of Fibrosis: Relative ADC

Quantification of fibrosis is conditioned by variability in the measurement process, as already reported in several articles [63–65]. To reduce variability, the possibility of calculating relative ADC has been considered. A recent article by Hong et al. evaluated sixty-seven patients affected by chronic B hepatitis and nine patients with focal liver lesions. They performed diffusion MR using different *b*-values and calculated liver ADCs and relative ADCs; the latter were obtained using splenic and renal parenchyma as references.

Namely, relative renal ADC was obtained according to the ratio between liver ADC and renal cortex ADC, whereas relative splenic ADC was calculated according to the ratio between liver ADC and splenic ADC [64]. All mean ADC values, absolute and relative, showed an inverse correlation with hepatic fibrosis and they were lower in increased fibrosis scores. The relative renal ADCs, obtained at *b* = 600 mm^2^/sec diffusion images, showed a strong correlation with degree of hepatic fibrosis, with an *r*-value of −0.697.

Moreover, Do et al. evaluated the contribution of a relative ADC measurement in the assessment of hepatic fibrosis [65]. The authors found that normalization of ADC, which means the ratio between liver ADC and spleen ADC, was more accurate than standard ADC measurement in the quantification of fibrosis [65]. For predicting moderate or advanced fibrosis (stage ≥ F2), ROC analysis reported an area under the curve of 0.864 using “normalized ADC,” greater than the value obtained using liver ADC (0.655) [65]. However, studies which have investigated the role of relative ADC in the measurement of liver fibrosis show many limitations. Mainly, they are based on a retrospective population study, sometimes limited in number of patients. In addition, relative measurement could be time-consuming, because radiologists need to calculate the ADC of two organs, and then to create a ratio between values.

## 3. Conclusions

Diffusion MR in liver fibrosis quantification cannot replace liver biopsy. Several studies published on this topic have not shown univocal results. Although many articles have demonstrated statistically significant results, ADC measurements are conditioned by overlap in the evaluation of different stages of fibrosis.

In addition, variability among measurement still represents a limit in diffusion imaging. Further investigation and analysis are needed to increase the reliability of the technique. A possible application for the future, in our opinion, could be the use of fibrosis monitoring in the management of the disease.
